# 

**Published:** 1925-01

**Authors:** 


					JANUARY, 1925.
V?l? XL1I. No. 155.
THE
BRISTOL
medico-chirurgical
AL
PUBLISHED UXDEIt TIIE AUSPICES OF
THE BRISTOL MEDICO-CHIRURGICAL
Editor: P. WATSON-WILLIAMSAM.D., W
\ ?v _ - o' /
WITH WHOM ARK ASSOCIATED X ? -V d 1^
J. LACY FIRTH, M.S., M.D., CAREY COOMBS, M.
E. W. HEY GROVES, M.S., F.R.C.S., B.Sc.
J. A. NIXON, C.M.G., M.D., Assistant Editor.
Editorial Stcretary: A. L. FLEMMING, M.B., B.Ch.
BRISTOL: J. W. ARROWSMITH LTD., n QUAY STREET.
ndon : j. Arrowsmith (London) Ltd., 6 Upper Bedford Place,
Russell Square.
Price Three Shillings net.

				

